# Influence of virtual monoenergetic reconstructions on coronary CT angiography-based fractional flow reserve with photon-counting detector CT: intra-individual comparison with energy-integrating detector CT

**DOI:** 10.1186/s13244-025-01927-5

**Published:** 2025-02-17

**Authors:** Giuseppe Tremamunno, Daniel Pinos, Emese Zsarnoczay, U. Joseph Schoepf, Milan Vecsey-Nagy, Chiara Gnasso, Nicola Fink, Dmitrij Kravchenko, Muhammad Taha Hagar, Joseph Griffith, Jim O’Doherty, Andrea Laghi, Tilman Emrich, Akos Varga-Szemes

**Affiliations:** 1 Department of Radiology and Radiological Science, Medical University of South Carolina, Charleston, SC USA; 2 Department of Medical Surgical Sciences and Translational Medicine, Sapienza University of Rome, Rome, Italy; 3 MTA-SE Cardiovascular Imaging Research Group, Department of Radiology, Medical Imaging Centre, Semmelweis University, Budapest, Hungary; 4 Heart and Vascular Center, Semmelweis University, Budapest, Hungary; 5 Experimental Imaging Center, IRCCS San Raffaele Scientific Institute, Milan, Italy; 6 Department of Radiology, University Hospital Munich, LMU Munich, Munich, Germany; 7 Department of Diagnostic and Interventional Radiology, University Hospital Bonn, Bonn, Germany; 8 Quantitative Imaging Laboratory Bonn (QILaB), Bonn, Germany; 9 Department of Diagnostic and Interventional Radiology, Medical Center—University of Freiburg, Freiburg, Germany; 10 Siemens Medical Solutions, Malvern, PA USA; 11 Department of Diagnostic and Interventional Radiology, University Medical Center of the Johannes Gutenberg-University Mainz, Mainz, Germany

**Keywords:** Computed tomography angiography, Energy-integrating detector, Fractional flow reserve, Photon-counting detector, Virtual monoenergetic image

## Abstract

**Objectives:**

This study aimed to assess the impact of the photon-counting detector (PCD)-CT-based virtual monoenergetic image (VMI) reconstruction keV levels on CT-based fractional flow reserve (CT-FFR), compared to the energy-integrating detector (EID)-CT.

**Methods:**

Patients undergoing clinically indicated coronary CT angiography (CCTA) on an EID-CT were prospectively enrolled for a research CCTA on a PCD-CT within 30 days. PCD-CT datasets were reconstructed at VMI levels of 45, 55, 70, and 90 keV. CT-FFR was obtained semiautomatically using an on-site machine learning algorithm by two readers. CT-FFR ≤ 0.80 was considered hemodynamically significant.

**Results:**

A total of 20 patients (63.3 ± 8.8 years; 13 men (65%) were included. Median CT-FFR values in the per-vessel analysis for PCD-CT scans were 0.86 (0.81–0.92) for 45 keV, 0.87 (0.80–0.93) for 55 keV, 0.85 (0.79–0.92) for 70 keV and 0.82 (0.76–0.89) for 90 keV, and 0.86 (0.71–0.93) for EID-CT. Comparison among different VMIs showed significant differences only for 45 vs. 90 keV (*p* < 0.001), and 55 vs. 90 keV (*p* < 0.001). No significant differences were found in the pairwise comparison between any VMI and EID-CT (all *p* > 0.05). PCD-CT at 70 keV showed the highest correlation (*r* = 0.83, *p* < 0.001), agreement (ICC: 0.90 (0.84–0.94)), and the lowest bias (mean bias −0.01; limits of agreement, 0.84/0.94) when compared to EID-CT.

**Conclusion:**

VMI reconstructions showed significant influence on CT-FFR values only at the extreme levels of the spectrum, while no significant differences were found in comparison with EID-CT. VMI at 70 keV demonstrates the highest correlation and agreement, with the lowest bias compared to EID-CT.

**Critical relevance statement:**

Evidence on novel spectral photon-counting detector (PCD)-CT’s impact on CT-fractional flow reserve (FFR) is limited; our results demonstrate the feasibility of CT-FFR using PCD-CT, showing no significant differences between various virtual monoenergetic images and energy-integrating detector (EID)-CT values

**Key Points:**

- The impact of spectral photon-counting detector (PCD)-CT on CT-derived fractional flow reserve (CT-FFR) is unclear.
- Spectral PCD-CT-based CT-FFR is feasible, differing only at extreme virtual monoenergetic image levels.
- CT-FFR from PCD-CT at 70 keV showed the strongest correlation with energy-integrating detector-CT.

**Graphical Abstract:**

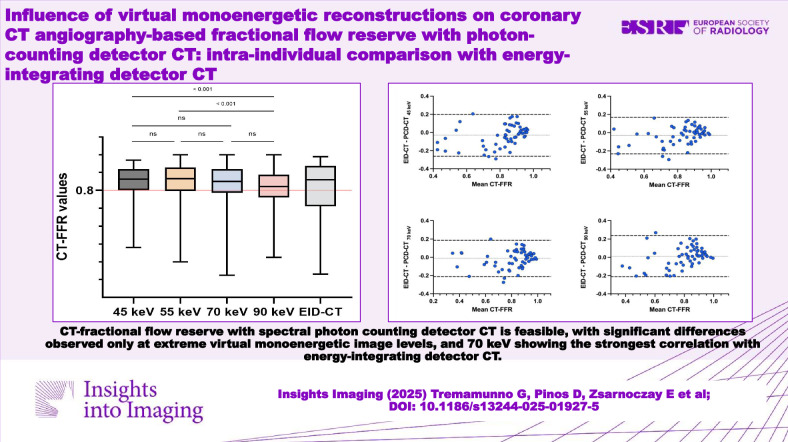

## Introduction

Coronary artery disease (CAD) remains the leading cause of morbidity and mortality worldwide [1, 2]. In recent years, advancements in CT technology have revolutionized the field of cardiovascular imaging, enabling coronary computed tomography angiography (CCTA) to play a cornerstone role and become a first-line imaging modality for CAD in patients with stable chest pain [3, 4]. Even though CCTA provides excellent anatomical information on coronary arteries, it has moderate specificity for the diagnosis of obstructive CAD, which can lead to unnecessary additional examinations [5, 6]. Invasive fractional flow reserve (iFFR) is considered the gold standard in the evaluation of lesion-specific functional significance [7]. However, it requires the acquisition of invasive coronary angiography, which is associated with potential complications (e.g., vessel dissection or perforation) [8]. CCTA-derived FFR (CT-FFR) has emerged as a promising non-invasive alternative for evaluating the functional significance of CAD, which uses computational fluid dynamics to calculate FFR values from CCTA images [9]. It has been validated against iFFR and showed the ability to predict lesion-specific ischemia and clinical outcomes in patients with CAD [10–12]. With the advent of artificial intelligence, it is now possible to compute on-site CT-FFR on physician-driven workstations [13].

Photon-counting detector (PCD)-CT, utilizing cadmium telluride for direct photon-to-electric signal conversion, can overcome the limitations of conventional energy-integrating detector (EID)-CT systems [14]. Several studies investigated the multiple advantages of PCD-CT and demonstrated increased contrast-to-noise ratio, decreased electronic noise, decreased blooming artifacts, and improved spatial resolution [14–19]. Monochromatic reconstructions from single-source kV switching dual-energy CCTA have been shown to improve coronary stent and coronary stenosis imaging compared to polychromatic reconstructions [20, 21]. Although PCD-CT enables the generation of virtual monoenergetic images (VMI), the impact of the different VMI keV levels on CT-FFR evaluation remains unclear.

Therefore, this study aimed to prospectively assess the impact of PCD-CT-based VMI reconstruction keV levels on CT-FFR and to compare them to EID-CT in patients undergoing CCTA on both systems.

## Materials and methods

### Study design and population

The local Institutional Review Board approved the protocol of this prospective, single-center, observational study, and all subjects signed written informed consent. Consecutive patients > 18 years of age referred for standard-of-care CCTA on an EID-CT system were recruited for a research PCD-CT scan within 30 days between July 2021 and March 2022. Exclusion criteria were contraindication for iodinated contrast media, pregnancy or ongoing lactation, reduced kidney function with a glomerular filtration rate under 45 mL/min/1.73 m^2^, inability to complete the informed consent form, and poor image quality precluding coronary artery segmentation. Furthermore, patients with prior coronary artery bypass grafting or percutaneous coronary intervention and coronary arteries affected by image artifacts were excluded from the CT-FFR analysis. Image artifacts included motion artifacts (cardiac, respiratory, and patient), contrast material related, inadequate bolus timing, and quantum mottle artifacts [22].

### CCTA acquisition and reconstruction

First, all patients underwent a clinical CCTA using a third-generation dual-source EID-CT (SOMATOM Force, Siemens Healthineers, Forchheim, Germany) according to clinical protocol. All pre-transcatheter aortic valve replacement scans and other CCTAs where the heart rate exceeded 80 beats per minute or arrhythmia was present, were performed using a retrospectively gated helical scan mode. In contrast, non-transcatheter aortic valve replacement candidates with a regular heart rate below 80 beats per minute were scanned using sequential mode. According to the standard clinical protocol, both tube voltage and tube current were automatically determined by the scanner at 90, 100, 110, or 130 kVp using CARE kV, depending on the patient’s body habitus and using CARE Dose4D (Siemens), respectively. Images from EID-CT were reconstructed with a slice thickness of 0.5 mm and an increment of 0.3 mm, using a Bv36 vascular kernel, a matrix of 512 × 512, and Advanced Modeled Iterative Reconstruction (ADMIRE) set to level 3.

Research CCTA was subsequently conducted on a first-generation clinical dual-source PCD-CT system (NAEOTOM Alpha; Siemens). The tube voltage was set to 120 kVp, and the tube current was manually adjusted to closely match the expected radiation dose (CTDIvol) with the clinical scans. Images from PCD-CT were reconstructed at a slice thickness of 0.6 mm and increment of 0.4 mm, with Bv44 kernel, a matrix of 512 × 512, and quantum iterative reconstruction (QIR) strength level 3. Initially, 40, 55, 70, and 100 keV VMI levels were reconstructed. However, after analyzing all reconstructions, CT-FFR calculations were not feasible at 40 and 100 keV in several cases. Consequently, instead of 40 and 100 keV, we reconstructed 45 and 90 keV, where we found CT-FFR analysis to be feasible. The CT-FFR prototype program had been designed to recognize specific characteristics on scans. The machine learning algorithm was trained with traditional data (80–120 kV) where extremely low and high VMI levels are not able to be processed. In the final analysis, VMI was reconstructed at 45, 55, 70, and 90 keV. Image acquisition parameters are summarized in Table 1.

**Table 1:** CCTA acquisition and reconstruction parameters

	EID-CT	PCD-CT
Tube potential (kVp)	90 /100 /110/130	120
Tube current (mAs)	262.5 (221.0–402.3)	Image quality level: 64
Rotation time (s)	0.25	0.25
Temporal resolution (ms)	66	66
Reconstruction energy threshold	Polychromatic	45, 55, 70, 90 keV
Iterative reconstruction (strength level)	ADMIRE (3)	QIR (3)
Reconstruction kernel	Bv36	Bv44
Slice thickness (mm)	0.5	0.6
Slice increment (mm)	0.3	0.4
Matrix size	512 × 512	512 × 512

Continuous variables are expressed as median (interquartile range)

*ADMIRE* advanced modeled iterative reconstruction, *CCTA* coronary CT angiography, *EID-CT* energy-integrating detector CT, *PCD-CT* photon-counting detector CT, *QIR* quantum iterative reconstruction

All patients received 0.4 mg of sublingual nitroglycerin. Additionally, patients with a heart rate above 70 beats per minute received 5 mg of intravenous metoprolol before the examination. The contrast administration strategy employed was the same for both CCTA acquisitions and followed the institutional protocols, using bolus tracking with an enhancement threshold of 150 HU in the descending aorta and a time delay of 8 s. The patients received a triphasic injection with a rate of 4 mL/s, including: (1) 50 mL of iopromide 350 mgI/mL (Ultravist, Bayer Healthcare, Leverkusen, Germany), (2) 20 mL of 50% mixture of contrast and saline, finally (3) 25 mL of saline.

### CT-FFR analysis

CT-FFR was computed from CCTA datasets using a machine learning algorithm [10, 23]. In brief, the algorithm was trained using a multilayer deep neural network architecture on a synthetically generated database of 12,000 coronary models with known 3-dimensional (3D) characteristics. A computational fluid dynamics (CFD) model was used to assess the FFR values for each coronary tree. The machine learning model was trained to calculate FFR based on the relationship between the anatomic features and the CFD-based FFR value. This method was previously validated against the CFD algorithm using an independent database of 87 patient-specific anatomical models derived from the CCTA compared to iFFR as a reference standard. The software system (cFFR version 3.5.1, Siemens, prototype software; syngo.via Frontier platform) was an offline, on-site standard desktop computer workstation solution allowing the physician-driven creation of a patient-specific anatomical model of the coronary system using a semi-automatic approach. Then, after accepting or correcting the luminal centerline and contour, the stenoses were marked, and the CT-FFR was computed, resulting in a color-coded 3D mesh of the coronary tree. The CT-FFR value approximately 2 cm distal to the stenosis (or distal end of the middle arterial segment if no stenosis) was recorded for the main vessels in the coronary system: left anterior descending, left circumflex, and right coronary arteries. CT-FFR values were measured at the same location for all four keV levels and the EID-CT dataset in each patient. The cut-off point for the analysis was set at 0.80, a value equal to or below that was considered abnormal and hemodynamically significant [24]. Patients with at least one vessel with a CT-FFR ≤ 0.80 were classified as hemodynamically significant, while a normal and hemodynamically non-significant classification required CT-FFR > 0.80 in all three vessels. CT-FFR calculations were performed by a reader with 3 years of experience in cardiovascular imaging and subsequently by a reader with 1 year of experience in cardiovascular imaging to assess the agreement between different CT-FFR values.

### Statistical analysis

Statistical analysis was performed using dedicated software (SPSS Statistics, version 27.0, IBM Corporation, and GraphPad Prism Version 8.4.2; GraphPad, San Diego, CA, USA). The Shapiro-Wilk test was used to test continuous data for normality. Normally distributed variables are reported as means ± standard deviations, and non-normally distributed variables are reported as medians with interquartile ranges. Analysis was performed on a per-patient and per-vessel basis. The per-patient CT-FFR was defined as the lowest CT-FFR value among the three vessels. Wilcoxon matched-pairs signed rank test was used to assess pairwise differences in CT-FFR values at different keV levels. Bonferroni correction was applied, and *p* = 0.05/6 = 0.008 was considered statistically significant for testing the four reconstructions. Wilcoxon matched-pairs signed rank test with a significant value of *p* = 0.05 was used to assess pairwise differences in CT-FFR between each VMI and EID-CT dataset. Spearman correlation coefficient (*r*) was used to assess the CT-FFR correlation between the four VMI levels and EID-CT dataset, and the correlation was displayed on scatterplots. The agreement between different CT-FFR values was evaluated using a two-way random-effects intraclass correlation coefficient (ICC) with the following interpretation: 0.0–0.3, lack of agreement; 0.31–0.5, weak; 0.51–0.7, moderate; 0.71–0.9, strong; and 0.91–1.00, very strong agreement. Bland–Altman plots were generated, illustrating the mean bias and limits of agreement (LoA) in CT-FFR between PCD-CT and EID-CT datasets. McNemar’s test was employed to assess the reclassification of hemodynamically significant/non-significant vessels and patients among the different reconstructions.

## Results

### Patient cohort

In total, 34 patients underwent CCTA on both EID and PCD-CT, from which 14 patients were excluded due to pacemaker implantation (*n* = 4), prior coronary artery bypass grafting (*n* = 3), software unable to automatically segment the luminal centerline at 45 keV (*n* = 2), at 90 keV (*n* = 2), at EID-CT (*n* = 2), and prior percutaneous coronary intervention (*n* = 1). The final cohort included 20 patients (63.3 ± 8.8 years; 13 men (65%)), and the median time between EID- and PCD-CT acquisitions was 5.5 days (1.8–13.6). Each patient had CT-FFR analysis in all three major coronary arteries. Patient characteristics are summarized in Table 2.

**Table 2:** Patient characteristics

	EID-CT	PCD-CT	*p*
Age (years)	63.3 ± 8.8		
Sex (male, %)	13 (65)		
Body height (cm)	172.7 ± 11.2		
Body weight (kg)	98.9 ± 32.9		
Body mass index (kg/m^2^)	32.5 ± 8.3		
Time between scans (days)	5.5 (1.8–13.6)		
Heart rate during CCTA (bpm)	69.3 ± 13.3	63.1 ± 12.1	0.13
CTDI_*vol*_ (mGy)	38.0 (24.6–61.1)	26.2 (18.2–40.1)	0.01
DLP (mGy∗cm)	452.8 (342.8–903.7)	368.7 (254.4–602.8)	0.049

Continuous variables are expressed as mean ± standard deviation, or median (interquartile range). Categorical variables are expressed as frequencies (percentages)

*bpm* beats per minute, *CCTA* coronary CT angiography, *CTDI* computed tomography dose index, *DLP* dose length product, *EID-CT* energy-integrating detector CT, *PCD-CT* photon-counting detector CT

### Per-vessel analysis

Per-vessel analysis was conducted on 60 vessels. Median CT-FFR values for PCD-CT scans at 45, 55, 70, and 90 keV were 0.86 (0.81–0.92), 0.87 (0.80–0.93), 0.85 (0.79–0.92), and 0.82 (0.76–0.89), respectively. Pairwise comparison of CT-FFR values between the different keV levels showed no significant difference for 45 vs. 55 keV (*p* = 0.81), 45 vs. 70 keV (*p* = 0.20), 55 vs. 70 keV (*p* = 0.09), and 70 vs. 90 keV (*p* = 0.02). However, a significant difference was found for 45 vs. 90 keV (*p* < 0.001), and 55 vs. 90 keV (*p* < 0.001).

**Fig. 1:**
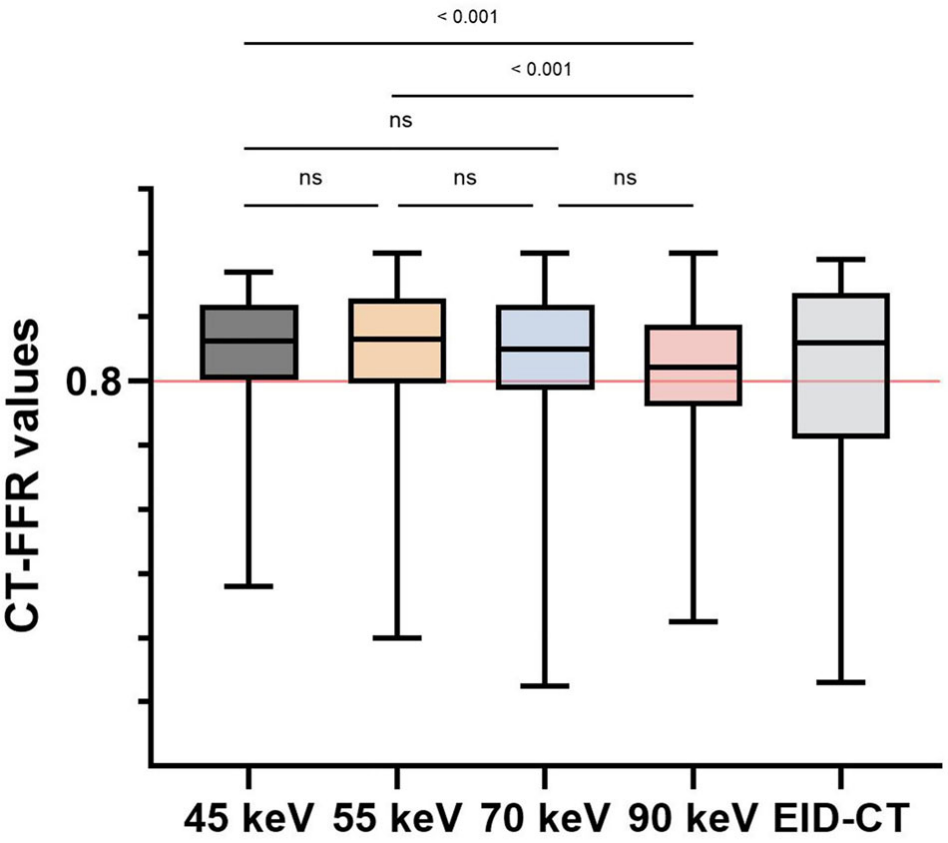
Box plot with line diagram shows the per-vessel CT angiography-derived fractional flow reserve (CT-FFR) comparison between photon-counting detector (PCD)-CT virtual monoenergetic image (VMI) at 45 keV, 55 keV, 70 keV, 90 keV and energy-integrating detector (EID)-CT

**Fig. 2:**
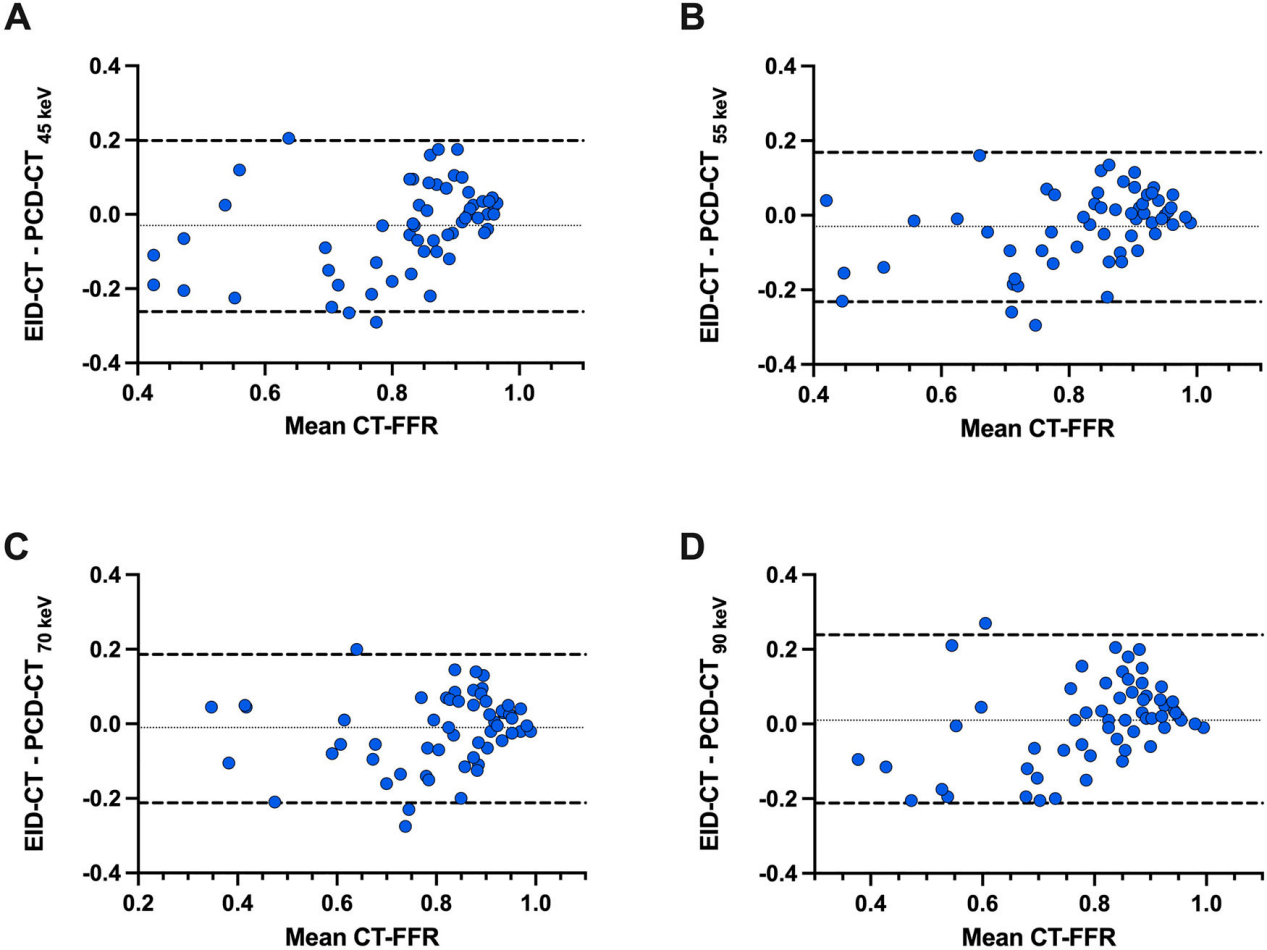
Bland–Altman plots show the per-vessel CT angiography-derived fractional flow reserve (CT-FFR) comparisons between energy-integrating detector (EID)-CT, and virtual monoenergetic image (VMI) at 45 keV (**A**), 55 keV (**B**), 70 keV (**C**), and 90 keV (**D**) from photon-counting detector (PCD)-CT

**Fig. 3:**
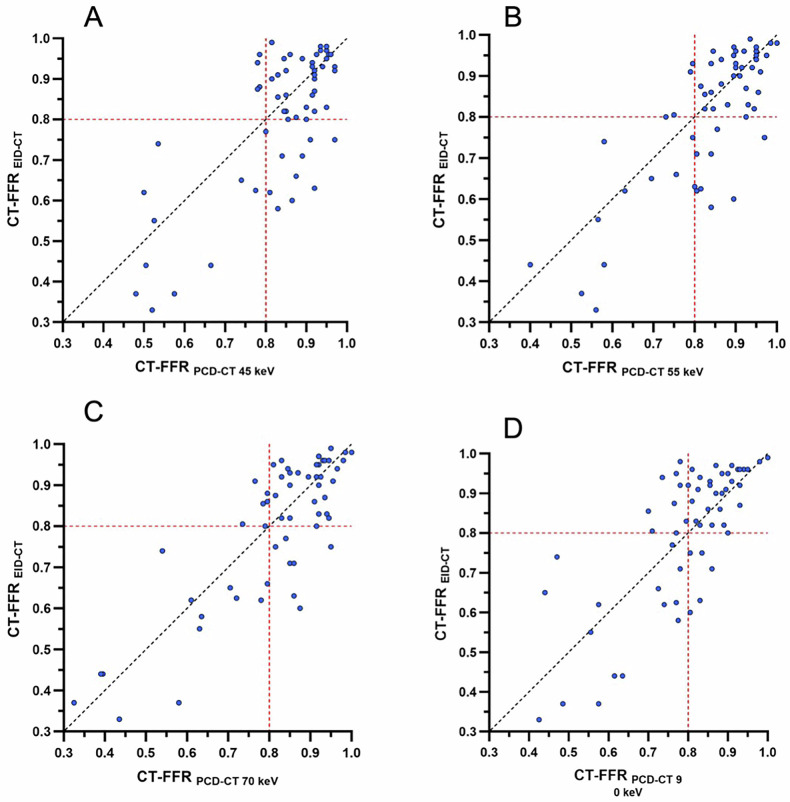
Scatterplots show the per-vessel CT angiography-derived fractional flow reserve (CT-FFR) comparisons between energy-integrating detector (EID)-CT and virtual monoenergetic image (VMI) at 45 keV (**A**), 55 keV (**B**), 70 keV (**C**), and 90 keV (**D**) from the photon-counting detector (PCD)-CT

The median per-vessel CT-FFR value for EID-CT was 0.86 (0.71–0.93). No significant differences were observed in the pairwise comparison between EID-CT and 45 keV (*p* = 0.08), 55 keV (*p* = 0.08), 70 keV (*p* = 0.45), and 90 keV (*p* = 0.44) (Fig. 1). CT-FFR values at all keV levels demonstrated a strong correlation and agreement with those derived from EID-CT (Table 3). CT-FFR at 70 keV demonstrated the highest correlation (*r* = 0.83, *p* < 0.001), agreement (ICC: 0.90 (0.84–0.94)), and the lowest bias (mean bias −0.01; LoA, −0.21/0.18) when compared to EID-CT-based CT-FFR. Figures 2 and 3 show the Bland–Altman plots and scatterplots among all VMI levels and EID-CT, respectively.

**Table 3:** Comparison of per-vessel CT-FFR values between EID-CT and different PCD-CT datasets

Dataset	CT-FFR	*p*^*a*^	*r*^a^	ICC (95% CI)^a^	Bias^a^	LoA^a^
EID-CT	0.86 (0.71–0.93)					
PCD-CT 45 keV	0.86 (0.81–0.92)	0.08	0.73 (0.59–0.83) *p* < 0.001	0.83 (0.71–0.89)	−0.03	−0.26/0.20
PCD-CT 55 keV	0.87 (0.80–0.93)	0.08	0.82 (0.71–0.89) *p* < 0.001	0.88 (0.80–0.93)	−0.03	−0.23/0.17
PCD-CT 70 keV	0.85 (0.79–0.92)	0.45	0.83 (0.73–0.89) *p* < 0.001	0.90 (0.84–0.94)	−0.01	−0.21/0.18
PCD-CT 90 keV	0.82 (0.76–0.89)	0.44	0.76 (0.63–0.85) *p* < 0.001	0.85 (0.75–0.91)	0.01	−0.21/0.23

CT-FFR values are median (25th and 75th percentiles)

*CT-FFR* CT angiography-derived fractional flow reserve, *EID-CT* energy-integrating detector CT, *ICC* intraclass correlation coefficient, *LoA* limits of agreement, *PCD-CT* photon-counting detector CT

^a^ Every PCD-CT dataset is compared to EID-CT, which was considered the reference

Per-vessel inter-reader agreement was strong to very strong for all comparisons: 45 keV (ICC = 0.92 (95% CI: 0.86–0.95)), 55 keV (0.89 (0.81–0.93)), 70 keV (0.87 (0.79–0.92)), 90 keV (0.92 (0.87–0.95)), and EID-CT (0.94 (0.90–0.96)).

### Per-patient analysis

The median per-patient (*n* = 20) CT-FFR values were as follows: 45 keV: 0.81 (0.76–0.85), 55 keV: 0.81 (0.70–0.84), 70 keV: 0.78 (0.68–0.81), 90 keV: 0.76 (0.67–0.81). Pairwise comparison of CT-FFR values between the different keV levels showed no significant difference for 45 vs. 55 keV (*p* = 0.27), 45 vs. 90 keV (*p* = 0.01), 55 vs. 70 keV (*p* = 0.06), 55 vs. 90 keV (*p* = 0.07), and 70 vs. 90 keV (*p* = 0.88); significant difference, however, was found for 45 vs. 70 keV (*p* = 0.007).

**Fig. 4:**
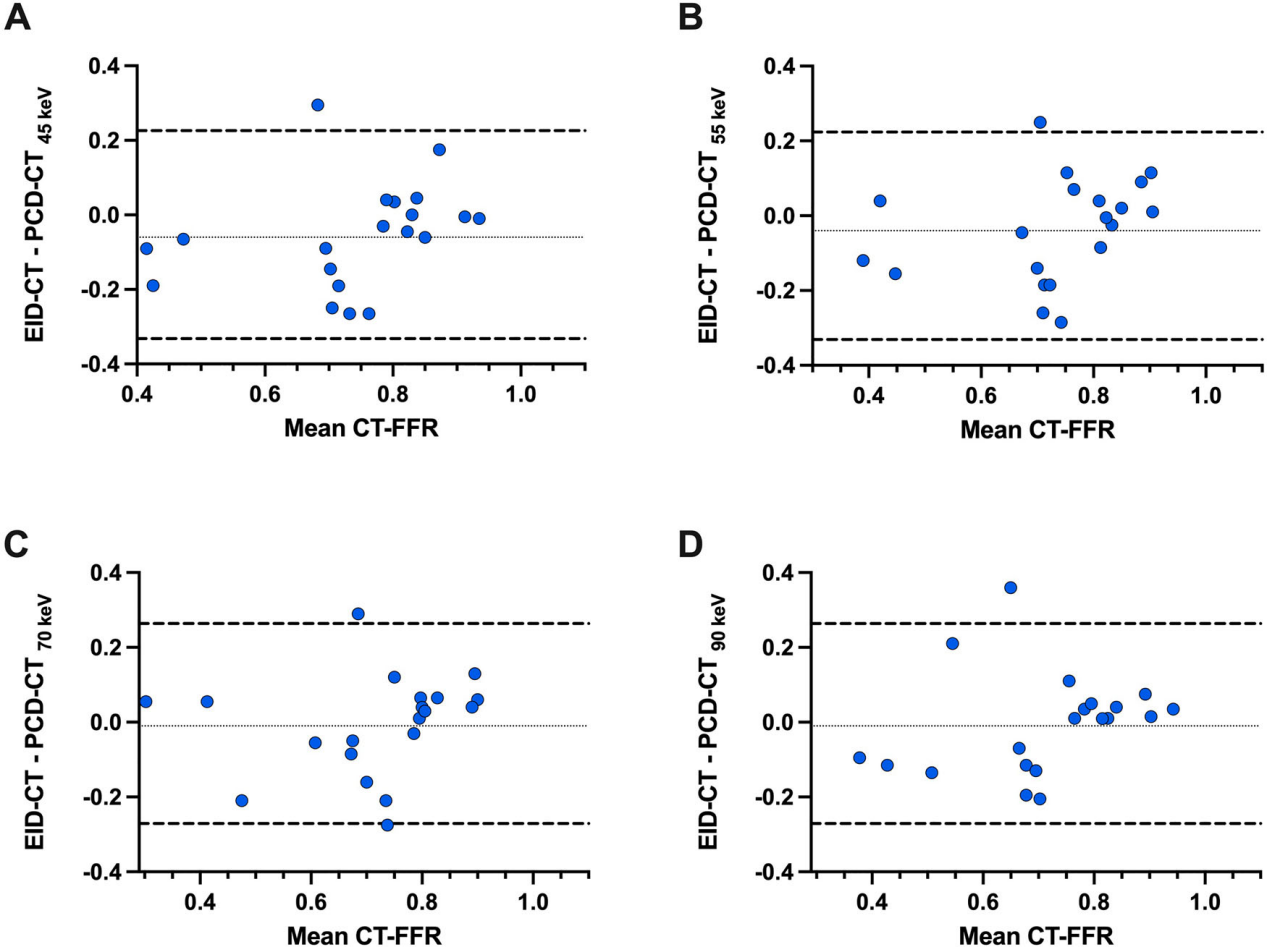
Bland–Altman plots show the per-patient CT angiography-derived fractional flow reserve (CT-FFR) comparisons between energy-integrating detector (EID)-CT and virtual monoenergetic image (VMI) at 45 keV (**A**), 55 keV (**B**), 70 keV (**C**), and 90 keV (**D**) from photon-counting detector (PCD)-CT

**Fig. 5:**
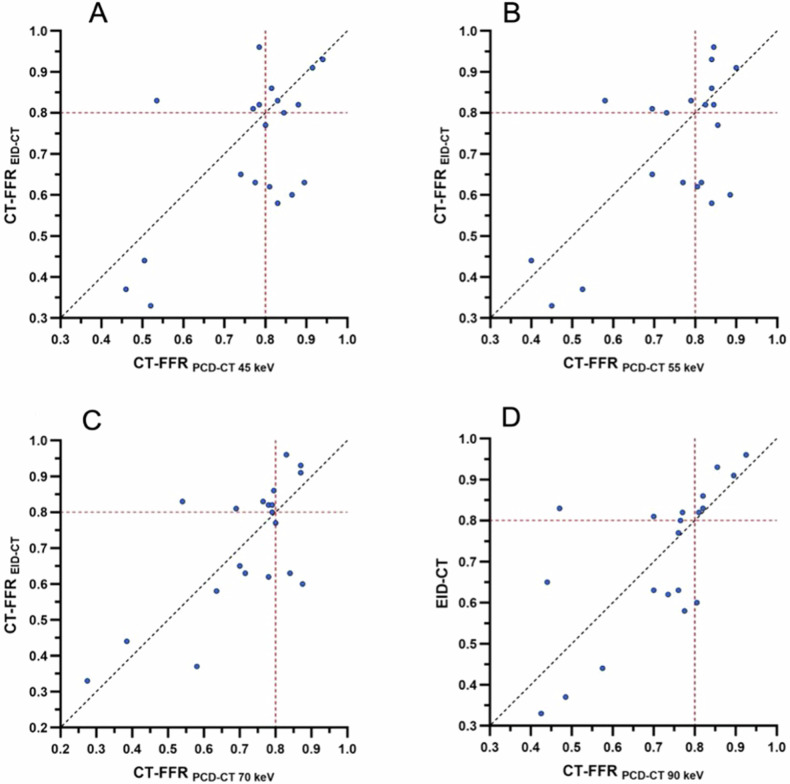
Scatterplots show the per-patient CT angiography-derived fractional flow reserve (CT-FFR) comparisons between energy-integrating detector (EID)-CT and virtual monoenergetic image (VMI) at 45 keV (**A**), 55 keV (**B**), 70 keV (**C**), and 90 keV (**D**) from photon-counting detector (PCD)-CT

The median per-patient CT-FFR value for EID-CT was 0.79 (0.62–0.83). No significant differences were observed in the pairwise comparison between EID-CT and 45 keV (*p* = 0.06), 55 keV (*p* = 0.26), 70 keV (*p* = 0.84) and 90 keV (*p* = 0.77). CT-FFR values at every keV demonstrated a strong correlation and agreement with CT-FFR values obtained from EID-CT (Table 4). The highest correlation (*r* = 0.71, *p* < 0.001), agreement (ICC: 0.82 (0.56–0.93)), and the lowest bias (mean bias −0.01; LoA, −0.27/0.26) was observed at 70 keV when compared to EID-CT. Figures 4 and 5 show the Bland–Altman plots and scatterplots across all VMI levels and EID-CT.

**Table 4:** Comparison of per-patient CT-FFR values between EID-CT and different PCD-CT datasets

Dataset	CT-FFR	*p*^*a*^	*r*^a^	ICC (95% CI)^a^	Bias^a^	LoA^a^
EID-CT	0.79 (0.62–0.83)					
PCD-CT 45 keV	0.81 (0.76–0.85)	0.06	0.64 (0.28–0.85) *p* = 0.002	0.77 (0.42–0.91)	−0.06	−0.33/0.22
PCD-CT 55 keV	0.81 (0.70–0.84)	0.26	0 0.67 (0.32–0.86) *p* = 0.001	0.79 (0.47–0.92)	−0.04	−0.31/0.23
PCD-CT 70 keV	0.78 (0.68–0.81)	0.84	0.71 (0.38–0.87) *p* < 0.001	0.82 (0.56–0.93)	−0.01	−0.27/0.26
PCD-CT 90 keV	0.76 (0.67–0.81)	0.77	0.68 (0.34–0.86) *p* < 0.001	0.80 (0.50–0.92)	−0.01	−0.27/0.26

CT-FFR values are median (25th and 75th percentiles)

*CT-FFR* CT angiography-derived fractional flow reserve, *EID-CT* energy-integrating detector CT, *ICC* intraclass correlation coefficient, *LoA* limits of agreement, *PCD-CT* photon-counting detector CT

^a^ Every PCD-CT dataset is compared to EID-CT, which was considered the reference

**Fig. 6:**
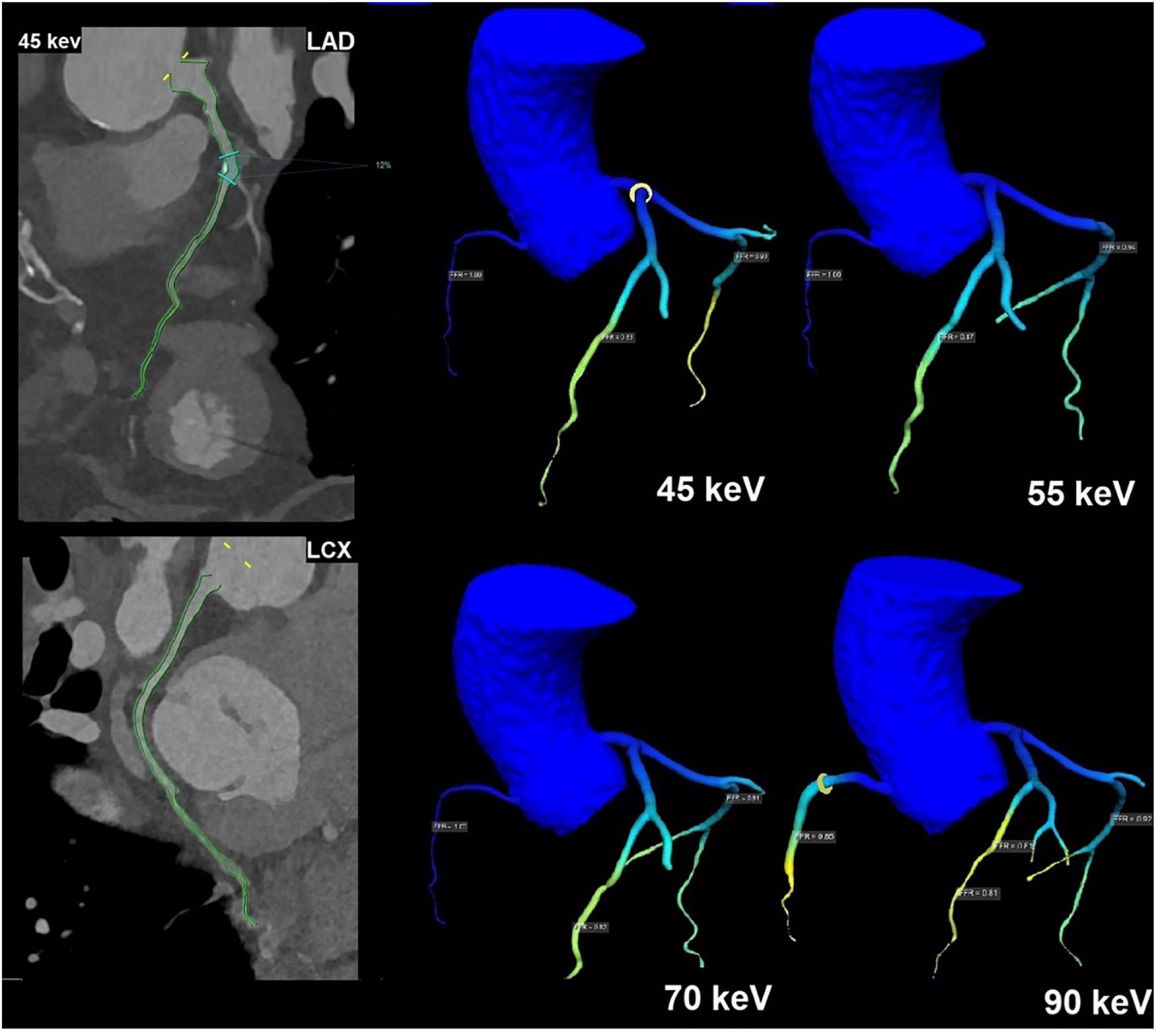
Image of a 66-year-old female patient with stenosis in the left anterior descending artery (LAD). Images are reconstructed at different virtual monoenergetic image (VMI) levels. Photon-counting detector (PCD)-CT images at 45 keV show a 12% LAD stenosis (marked by blue lines) by the machine learning–based software (cFFR prototype), and the final CT angiography-derived fractional flow reserve (CT-FFR) result. CT-FFR calculation shows no hemodynamical significance for the LAD (45 keV: 0.88, 55 keV: 0.87, 70 keV: 0.83, 90 keV: 0.81). No evidence of stenosis was found in the left circumflex artery and right coronary artery

Figure 6 demonstrates a case example comparing CT-FFR at different keV.

Per-patient inter-reader agreement was very strong for all comparisons: 45 keV (ICC = 0.90 (0.74–0.96)), 55 keV (0.93 (0.82–0.97)), 70 keV (0.92 (0.79–0.97)), 90 keV (0.95 (0.88–0.98)), and EID-CT (0.94 (0.85–0.98)).

### Inter-scanner reclassification

The per-vessel analysis revealed no significant differences in the distribution of hemodynamically significant lesions between EID-CT and all VMI series from PCD-CT. In total, 22 (36.7%) hemodynamically significant stenoses were identified in the EID-CT datasets compared to 15 (25%) at 45 keV (*p* = 0.118), 16 (26.7%) at 55 keV (*p* = 0.146), 19 (31.7%) at 70 keV (*p* = 0.581), and 25 (41.7%) at 90 keV (*p* = 0.607). In a per-patient analysis, performing the CT-FFR analysis on PCD-CT resulted in the reclassification into the hemodynamically significant group of 4 patients at 45 keV, 3 patients at 55 keV, 5 patients at 70 keV, and 3 patients at 90 keV, who were otherwise classified as hemodynamically non-significant by EID-CT. However, the distribution of patients assigned to the significant group in the inter-scanner comparison did not result in statistically significant differences, as detailed in Table 5.

**Table 5:** Per-vessel and per-patient analysis for CT-FFR values with the distribution of hemodynamically significant lesions

	EID-CT		PCD-CT 45 keV			PCD-CT 55 keV			PCD-CT 70 keV			PCD-CT 90 keV		
	≤ 0.80^a^	> 0.80^a^	≤ 0.80	> 0.80	p*	≤ 0.80	> 0.80	p*	≤ 0.80	> 0.80	p*	≤ 0.80	> 0.80	p*
Per-vessel														
LAD	10 (50.0)	10 (50.0)	7 (35.0)	13 (65.0)	0.45	5 (25.0)	15 (75.0)	0.06	7 (35.0)	13 (65.0)	0.45	9 (45.0)	11 (55.0)	1.00
LCX	5 (25.0)	15 (75.0)	3 (15.0)	17 (85.0)	0.63	3 (15.0)	17 (85.0)	0.63	3 (15.0)	17 (85.0)	0.50	8 (40.0)	12 (60.0)	0.45
RCA	7 (35.0)	13 (65.0)	5 (25.0)	15 (75.0)	0.63	8 (40.0)	12 (60.0)	1.00	9 (45.0)	11 (55.0)	0.63	8 (40.0)	12 (60.0)	1.00
Total	22 (36.7)	38 (63.3)	15 (25.0)	45 (75.0)	0.12	16 (26.7)	44 (73.3)	0.15	19 (31.7)	41 (68.3)	0.58	25 (41.7)	35 (58.3)	0.61
Per-patient														
	11 (55.0)	9 (45.0)	10 (50.0)	10 (50.0)	1.00	9 (45.0)	11 (55.0)	0.73	15 (75.0)	5 (25.0)	0.29	13 (65.0)	7 (35.0)	0.63

Values are *n* (%)

* Differences in the distribution between every PCD-CT dataset and EID-CT are tested with McNemar’s test

*CT-FFR* CT angiography-derived fractional flow reserve, *EID-CT* energy-integrating detector CT, *LAD* left anterior descending artery, *LCX* left circumflex artery, *PCD-CT* photon-counting detector CT, *RCA* right coronary artery

^a^ The values ≤ 0.80 and > 0.80 represent CT-FFR measurements

## Discussion

This study investigated the impact of PCD-CT-based VMI reconstructions on CT-FFR values, comparing them with EID-CT, in a cohort of patients undergoing CCTA on both systems. The major findings are as follows: (a) comparison with different VMI reconstructions showed a statistically significant difference only at the extreme levels of the spectrum in a per-vessel analysis, with a slight trend of lower CT-FFR values at higher keV levels. (b) No significant differences were found when comparing each PCD-CT VMI level with EID-CT. (c) VMI at 70 keV level demonstrated the strongest correlation and agreement, with the least bias compared to conventional EID-CT.

CCTA is currently considered a viable non-invasive alternative for evaluating CAD in patients with stable and acute chest pain without known CAD [25, 26]. However, CCTA is limited in the anatomical assessment of calcified coronary stenoses and does not evaluate their functional hemodynamic significance, which is crucial for intermediate stenoses. According to clinical guidelines, the calculation of CT-FFR can be useful for intermediate-risk patients with coronary artery stenosis of 40 to 90% [25, 27]. Compared to the anatomical interpretation of CCTA, CT-FFR offers both anatomical and functional assessments, accurately identifies ischemia-causing lesions, and provides superior diagnostic accuracy to guide decisions on coronary revascularization [28–31].

However, most CT-FFR studies have been conducted using EID-CT systems and share a common limitation [32, 33]. Gao et al [34] found in a large multicenter study that CT-FFR’s diagnostic performance in the gray zone (FFR values between 0.75 and 0.80) decreases, particularly with calcified plaques. Similar findings were discussed by Rifai et al [35], who stated that the accuracy of CT-FFR in detecting stenosis with iFFR ≤ 0.80 was 74% in patients with high coronary calcium scores, compared to 83%–85% in those with low to mid coronary calcium scores. This discrepancy may be due to calcium blooming and overestimation of luminal stenosis affecting CT-FFR values [33, 35].

In this scenario, PCD-CT represents an emerging technology that has multiple advantages over EID-CT owing to its unique method of X-ray photon detection. The implementation of PCD-CT in clinical practice holds promise for addressing the problem of overestimation of CAD lesions. This is particularly evident with the improved iodine contrast-to-noise ratio, radiation dose efficiency, reduced calcium blooming artifacts, and provision of higher spatial resolution, which could optimize coronary arteries evaluation [16, 18, 36, 37]. Wolf et al demonstrated that with PCD-CT, the reduction of blooming artifacts and the measurement of coronary calcium exhibit higher accuracy compared to EID-CT when compared to the ground truth [38]. Moreover, PCD-CT seemed to be less susceptible to calcium blooming even in the assessment of coronary stenosis, resulting in lower average percentage diameter stenosis values compared to EID-CT, while still demonstrating a high inter-scanner correlation [39]. At present, there is a lack of evidence regarding the influence of the novel spectral technology of PCD-CT on CT-FFR; our study aims to fill this gap by including a cohort of patients who underwent CCTA with both scanners in a short time frame to assess the impact of different VMIs on CT-FFR and compare them with EID-CT, considered as the reference.

Our findings confirm the feasibility of CT-FFR computation with PCD-CT, as evaluated by Zsarnoczay et al, as no significant differences were observed between the different VMIs, and the values obtained from EID-CT [40]. At the lowest VMI levels, we measured higher CT-FFR values with a decreasing tendency at higher VMI levels in both per-vessel and per-patient analysis; in the per-vessel analysis, we found a significant difference in the CT-FFR measurements when comparing 45 vs. 90 keV and 55 vs. 90 keV, which represent the extreme values of the examined spectrum. The higher CT-FFR values at lower VMI might be explained by the greater vessel attenuation, contrast resolution, higher contrast-to-noise ratio, and improved vessel sharpness, resulting in enhanced image quality as assessed by Sartoretti et al [41]. The associated increase in image noise resulting from the use of low keV was addressed by the use of QIR at level 3 [42]. Enhanced vessel sharpness at lower VMI levels is partly due to better vascular contrast at lower energy levels. At lower keV, the attenuation difference between neighboring tissue and iodinated contrast in the vessel lumen increases, increasing objective image sharpness. Conversely, at higher VMI levels, the contrast-to-noise ratio decreases, likely resulting in lower CT-FFR values. Additionally, this difference could be attributed to the effect of different VMIs on stenosis assessment; while lower keV levels generally offer improved visualization of small vessels and plaque morphology, higher keV levels might be more effective for evaluating heavily calcified lesions due to reduced blooming artifacts [43–45]. Our findings suggest that the greatest agreement and correlation in CT-FFR values between EID-CT and different VMIs from PCD-CT occur at 70 keV, which represents a midpoint in the examined spectrum. However, further studies are needed to thoroughly assess the accuracy of CT-FFR measurements at different VMI levels from PCD-CT, using invasive reference as a benchmark. Additionally, it would be intriguing to explore the accuracy of PCD-CT-based FFR at various VMI levels while considering different plaque compositions and patient characteristics to provide personalization of imaging protocols.

The following limitations merit consideration. Firstly, the number of patients included in this study can be considered limited; nevertheless, what distinguishes this study is the incorporation of patients who underwent CCTA with the two distinct CT systems within a brief period, eliminating several potential confounders in the inter-scanner comparison. In spite of the strict study design, the limited number of enrolled patients may limit the generalizability of the results. Studies involving larger patient populations are warranted to confirm the effect of different VMI levels on CT-FFR. Second, we did not compare our results to iFFR; additional studies investigating CT-FFR with PCD-CT should include iFFR as a ground truth. Third, we evaluated only the effect of limited VMI levels on CT-FFR measurements, where the analysis performed by the machine learning algorithm was feasible. Furthermore, the effect of different reconstruction parameters, for example kernel and QIR levels, were not assessed. Moreover, the potential clinical implications and prognostic values of PCD-CT-based CT-FFR values remain unknown, and future prospective studies are necessary to evaluate long-term outcomes. Furthermore, during the planning phase of the study, PCD-CT ultrahigh-resolution technology was not available at our institution, and thus its feasibility was not evaluated. Future studies should analyze the impact of increased spatial resolution on CT-FFR. Lastly, in light of the fact that the majority of contemporary CT-FFR solutions employ the absolute value and spatial distribution of CT numbers as input for coronary detection and extraction, it can be posited that the extreme HU values observed at the lowest and highest VMI levels currently still present a challenge for alternative software as well, to a potentially even higher degree.

In conclusion, our investigation highlighted significant differences among VMI reconstructions at the extreme levels of the spectrum, indicating a tendency towards decreased CT-FFR values with higher keV levels. Conversely, the comparison between each VMI level from PCD-CT and EID-CT revealed no significant variances and the 70 keV levels demonstrated superior correlation, agreement, and minimal bias when compared to conventional EID-CT. Further research and clinical validation are necessary to establish optimal imaging protocols and broaden the applicability of the PCD-CT approach in routine clinical practice.

## Data Availability

The datasets used and/or analyzed during the current study are available from the corresponding author upon reasonable request.

## Abbreviations

CAD: Coronary artery disease
CCTA: Coronary CT angiography
EID-CT: Energy-integrating detector CT
FFR: Fractional flow reserve
ICC: Intraclass correlation coefficient
iFFR: Invasive fractional flow reserve
PCD-CT: Photon-counting detector CT
VMI: Virtual monoenergetic image

## References

[1] Ralapanawa U, Sivakanesan R (2021) Epidemiology and the magnitude of coronary artery disease and acute coronary syndrome: a narrative review. J Epidemiol Glob Health 11:169 33605111 10.2991/jegh.k.201217.001 PMC8242111

[2] Malakar AK, Choudhury D, Halder B, Paul P, Uddin A, Chakraborty S (2019) A review on coronary artery disease, its risk factors, and therapeutics. J Cell Physiol 234:16812–16823 30790284 10.1002/jcp.28350

[3] Budoff MJ, Dowe D, Jollis JG et al (2008) Diagnostic performance of 64-multidetector row coronary computed tomographic angiography for evaluation of coronary artery stenosis in individuals without known coronary artery disease: results from the prospective multicenter ACCURACY (Assessment by Coronary Computed Tomographic Angiography of Individuals Undergoing Invasive Coronary Angiography) trial. J Am Coll Cardiol 52:1724–1732 19007693 10.1016/j.jacc.2008.07.031

[4] Miller JM, Rochitte CE, Dewey M et al (2008) Diagnostic performance of coronary angiography by 64-row CT. N Engl J Med 359:2324–2336 19038879 10.1056/NEJMoa0806576

[5] Abdelrahman KM, Chen MY, Dey AK et al (2020) Coronary computed tomography angiography from clinical uses to emerging technologies: JACC state-of-the-art review. J Am Coll Cardiol 76:1226–1243 32883417 10.1016/j.jacc.2020.06.076 PMC7480405

[6] Parikh R, Patel A, Lu B, Senapati A, Mahmarian J, Chang SM (2020) Cardiac computed tomography for comprehensive coronary assessment: beyond diagnosis of anatomic stenosis. Methodist DeBakey Cardiovasc J 16:77 32670467 10.14797/mdcj-16-2-77 PMC7350823

[7] Stegehuis VE, Wijntjens GW, Piek JJ, Van De Hoef TP (2018) Fractional flow reserve or coronary flow reserve for the assessment of myocardial perfusion. Curr Cardiol Rep 20:77 10.1007/s11886-018-1017-4 PMC6061210 30046914

[8] Khalid N, Pandey Y, Khalid U et al (2021) Modes of failure with fractional flow reserve guidewires: insights from the manufacturer and user facility device experience database. World J Cardiol 13:223–229 34367506 10.4330/wjc.v13.i7.223 PMC8326154

[9] Budoff M, Nakansihi R (2016) Noninvasive FFR derived from coronary CT angiography in the management of coronary artery disease: technology and clinical update. Vasc Health Risk Manag. 10.2147/vhrm.s79632:269 10.2147/VHRM.S79632 PMC4922813 27382296

[10] Itu L, Rapaka S, Passerini T et al (2016) A machine-learning approach for computation of fractional flow reserve from coronary computed tomography. J Appl Physiol 121:42–52 27079692 10.1152/japplphysiol.00752.2015

[11] Coenen A, Kim YH, Kruk M et al (2018) Diagnostic accuracy of a machine-learning approach to coronary computed tomographic angiography-based fractional flow reserve: result from the MACHINE consortium. Circ Cardiovasc Imaging 11:e007217 29914866 10.1161/CIRCIMAGING.117.007217

[12] Tesche C, De Cecco CN, Albrecht MH et al (2017) Coronary CT angiography-derived fractional flow reserve. Radiology 285:17–33 28926310 10.1148/radiol.2017162641

[13] Renker M, Schoepf UJ, Wang R et al (2014) Comparison of diagnostic value of a novel noninvasive coronary computed tomography angiography method versus standard coronary angiography for assessing fractional flow reserve. Am J Cardiol 114:1303–1308 25205628 10.1016/j.amjcard.2014.07.064

[14] Zsarnóczay E, Varga-Szemes A, Emrich T et al (2023) Characterizing the Heart and the Myocardium With Photon-Counting CT. Invest Radiol. 10.1097/rli.0000000000000956 10.1097/RLI.0000000000000956 36822653

[15] Flohr T, Schmidt B (2023) Technical basics and clinical benefits of photon-counting CT. Invest Radiol 58:441–450 10.1097/RLI.0000000000000980 PMC10259209 37185302

[16] Mergen V, Sartoretti T, Baer-Beck M et al (2022) Ultra-high-resolution coronary CT angiography with photon-counting detector CT: feasibility and image characterization. Invest Radiol. 10.1097/rli.0000000000000897 10.1097/RLI.0000000000000897 PMC10184822 35640019

[17] Rajagopal JR, Farhadi F, Richards T et al (2021) Evaluation of coronary plaques and stents with conventional and photon-counting CT: benefits of high-resolution photon-counting CT. Radio Cardiothorac Imaging 3:e210102 10.1148/ryct.2021210102 PMC8581588 34778782

[18] Zsarnoczay E, Fink N, Schoepf UJ et al (2023) Ultra-high resolution photon-counting coronary CT angiography improves coronary stenosis quantification over a wide range of heart rates—a dynamic phantom study. Eur J Radiol 161:110746 36821957 10.1016/j.ejrad.2023.110746

[19] Kreisler B (2022) Photon counting detectors: concept, technical challenges, and clinical outlook. Eur J Radiol 149:110229 35278927 10.1016/j.ejrad.2022.110229

[20] Stehli J, Clerc OF, Fuchs TA et al (2016) Impact of monochromatic coronary computed tomography angiography from single-source dual-energy CT on coronary stenosis quantification. J Cardiovasc Comput Tomogr 10:135–140 26754621 10.1016/j.jcct.2015.12.008

[21] Stehli J, Fuchs TA, Singer A et al (2015) First experience with single-source, dual-energy CCTA for monochromatic stent imaging. Eur Heart J Cardiovasc Imaging 16:507–512 25525062 10.1093/ehjci/jeu282

[22] Leipsic J, Yang TH, Thompson A et al (2014) CT angiography (CTA) and diagnostic performance of noninvasive fractional flow reserve: results from the determination of fractional flow reserve by anatomic CTA (DeFACTO) study. AJR Am J Roentgenol 202:989–994 24758651 10.2214/AJR.13.11441

[23] Tesche C, Otani K, De Cecco CN et al (2020) Influence of coronary calcium on diagnostic performance of machine learning CT-FFR: results from MACHINE registry. JACC Cardiovasc Imaging 13:760–770 31422141 10.1016/j.jcmg.2019.06.027

[24] Adjedj J, De Bruyne B, Floré V et al (2016) Significance of intermediate values of fractional flow reserve in patients with coronary artery disease. Circulation 133:502–508 26733607 10.1161/CIRCULATIONAHA.115.018747

[25] Gulati M, Levy PD, Mukherjee D et al (2021) 2021 AHA/ACC/ASE/CHEST/SAEM/SCCT/SCMR Guideline for the Evaluation and Diagnosis of Chest Pain. J Am Coll Cardiol 78:e187–e285 34756653 10.1016/j.jacc.2021.07.053

[26] Narula J, Chandrashekhar Y, Ahmadi A et al (2021) SCCT 2021 Expert Consensus Document on Coronary Computed Tomographic Angiography: a report of the Society of Cardiovascular Computed Tomography. J Cardiovasc Comput Tomogr 15:192–217 33303384 10.1016/j.jcct.2020.11.001 PMC8713482

[27] Patel MR, Nørgaard BL, Fairbairn TA et al (2020) 1-year impact on medical practice and clinical outcomes of FFR_(CT)_: The ADVANCE Registry. JACC Cardiovasc Imaging 13:97–105 31005540 10.1016/j.jcmg.2019.03.003

[28] Kruk M, Wardziak Ł, Demkow M et al (2016) Workstation-based calculation of CTA-based FFR for intermediate stenosis. JACC Cardiovasc Imaging 9:690–699 26897667 10.1016/j.jcmg.2015.09.019

[29] Min JK, Leipsic J, Pencina MJ et al (2012) Diagnostic accuracy of fractional flow reserve from anatomic CT angiography. JAMA 308:1237–1245 22922562 10.1001/2012.jama.11274 PMC4281479

[30] Douglas PS, Pontone G, Hlatky MA et al (2015) Clinical outcomes of fractional flow reserve by computed tomographic angiography-guided diagnostic strategies vs. usual care in patients with suspected coronary artery disease: the prospective longitudinal trial of FFR(CT): outcome and resource impacts study. Eur Heart J 36:3359–3367 26330417 10.1093/eurheartj/ehv444 PMC4677273

[31] Fairbairn TA, Nieman K, Akasaka T et al (2018) Real-world clinical utility and impact on clinical decision-making of coronary computed tomography angiography-derived fractional flow reserve: lessons from the ADVANCE Registry. Eur Heart J 39:3701–3711 30165613 10.1093/eurheartj/ehy530 PMC6215963

[32] Nørgaard BL, Leipsic J, Gaur S et al (2014) Diagnostic performance of noninvasive fractional flow reserve derived from coronary computed tomography angiography in suspected coronary artery disease: The NXT Trial (Analysis of Coronary Blood Flow Using CT Angiography: Next Steps). J Am Coll Cardiol 63:1145–1155 24486266 10.1016/j.jacc.2013.11.043

[33] Rajiah P, Cummings KW, Williamson E, Young PM (2022) CT fractional flow reserve: a practical guide to application, interpretation, and problem solving. Radiographics 42:340–358 35119968 10.1148/rg.210097

[34] Gao Y, Zhao N, Song L et al (2022) Diagnostic performance of CT FFR with a new parameter optimized computational fluid dynamics algorithm from the CT-FFR-CHINA Trial: characteristic analysis of gray zone lesions and misdiagnosed lesions. Front Cardiovasc Med 9:819460 35391840 10.3389/fcvm.2022.819460 PMC8980684

[35] Al Rifai M, Ahmed AI, Alahdab F, Al-Mallah MH (2022) Clinical utility of coronary artery computed tomography angiography—what we know and what’s new? Prog Cardiovasc Dis 75:12–20 36336026 10.1016/j.pcad.2022.10.013

[36] Fink N, Zsarnoczay E, Schoepf UJ et al (2023) Radiation dose reduction for coronary artery calcium scoring using a virtual noniodine algorithm on photon-counting detector computed-tomography phantom data. Diagnostics 13:1540 37174932 10.3390/diagnostics13091540 PMC10177425

[37] Pinos D, Griffith J, Emrich T et al (2023) Intra-individual comparison of image quality of the coronary arteries between photon-counting detector and energy-integrating detector CT systems. Eur J Radiol 166:111008 37542817 10.1016/j.ejrad.2023.111008

[38] Wolf EV, Halfmann MC, Schoepf UJ et al (2023) Intra-individual comparison of coronary calcium scoring between photon counting detector- and energy integrating detector-CT: Effects on risk reclassification. Front Cardiovasc Med 9:1053398 10.3389/fcvm.2022.1053398 PMC9892711 36741832

[39] Wolf E, Gnasso C, Schoepf J et al (2023) Intra-individual comparison of coronary artery stenosis measurements between energy-integrating detector CT and photon-counting detector CT. Imaging 15:61–68

[40] Zsarnoczay E, Pinos D, Schoepf UJ et al (2024) Intra-individual comparison of coronary CT angiography-based FFR between energy-integrating and photon-counting detector CT systems. Int J Cardiol 399:131684 38151162 10.1016/j.ijcard.2023.131684

[41] Sartoretti T, McDermott M, Mergen V et al (2023) Photon-counting detector coronary CT angiography: impact of virtual monoenergetic imaging and iterative reconstruction on image quality. Br J Radiol 96:20220466 36633005 10.1259/bjr.20220466 PMC9975359

[42] Vecsey-Nagy M, Varga-Szemes A, Schoepf UJ et al (2024) Ultra-high resolution coronary CT angiography on photon-counting detector CT: bi-centre study on the impact of quantum iterative reconstruction on image quality and accuracy of stenosis measurements. Eur J Radiol 176:111517 38805884 10.1016/j.ejrad.2024.111517

[43] Hickethier T, Baeßler B, Kroeger JR et al (2017) Monoenergetic reconstructions for imaging of coronary artery stents using spectral detector CT: In-vitro experience and comparison to conventional images. J Cardiovasc Computed Tomogr 11:33–39 10.1016/j.jcct.2016.12.005 28096049

[44] Maaß C, Baer M, Kachelrieß M (2009) Image-based dual energy CT using optimized precorrection functions: a practical new approach of material decomposition in image domain. Med Phys 36:3818–3829 19746815 10.1118/1.3157235

[45] Wolf EV, Halfmann MC, Varga-Szemes A et al (2024) Photon-counting detector CT virtual monoenergetic images for coronary artery stenosis quantification: phantom and in vivo evaluation. AJR Am J Roentgenol 222:e2330481 38197760 10.2214/AJR.23.30481

